# Economic burden of type 2 diabetes management in France according to clinical characteristics

**DOI:** 10.1111/dom.70586

**Published:** 2026-03-09

**Authors:** Bruno Guerci, Christèle Blanc‐Bisson, Eric Vicaut, Gérard De Pouvourville, Bruno Detournay, Corinne Emery, Isabelle Bureau, Taos Ihaddadene‐Salzgeber, Fleur Levrat‐Guillen, Jean‐Pierre Riveline

**Affiliations:** ^1^ Department of Endocrinology‐Diabetology‐Nutrition CHRU Nancy, Brabois Hospitals, University of Lorraine Vandoeuvre‐les‐Nancy France; ^2^ Département de Médecine Générale Bordeaux University Bordeaux France; ^3^ URC HUSLS Lariboisière Fernand Widal Hospital Paris France; ^4^ Department of Economics ESSEC Business School Paris France; ^5^ Health Economics, Statistics Department CEMKA Bourg‐La‐Reine France; ^6^ Global HEOR (Health Economics and Outcomes Research) Abbott France Rungis France; ^7^ Global HEOR (Health Economics and Outcomes Research) Abbott Ltd Maidenhead UK; ^8^ Department of Diabetology and Endocrinology, Lariboisiere Hospital Assistance Publique – Hopitaux de Paris Paris France; ^9^ Unite INSERM U1138 Immunity and Metabolism in Diabetes ImMeDiab Team Institut Necker Enfants Malades and Université de Paris Cité Paris France

**Keywords:** costs, France, real‐world, retrospective study, treatment, type 2 diabetes

## Abstract

**Aim:**

We sought to investigate the treatment, medication patterns, and economic burden of type 2 diabetes (T2D) in France.

**Materials and Methods:**

This was a descriptive retrospective cross‐sectional study of a representative sample of adults in the national healthcare system claims database in 2022. Patients with T2D were compared with a propensity‐score‐matched cohort without diabetes.

**Results:**

In total, 80 127 patients with T2D (mean age, 68.4 years; 54.5% men; receiving diabetes treatment, 66 891) were matched to 237 607 controls. Insulin‐containing regimens were prescribed to 20.3% of treated patients; 40.0%, 24.8%, and 13.0% were receiving non‐insulin monotherapy, dual therapy and triple therapy, respectively. Insulin use was more common in overseas departments than in metropolitan France (25.5% vs. 20.0%). Glucagon‐like peptide 1 receptor agonists were prescribed to 17.0% of treated patients. Continuous glucose monitoring prescriptions were recorded in the last quarter of 2022 for 5.6% of treated patients (of whom 94.0% were using insulin). Overall, costs were higher for patients with T2D than for controls (6614€ vs. 3797€), and for patients in overseas departments, compared with metropolitan France (7210€ vs. 6484€).

**Conclusions:**

The cost of treating patients with T2D is almost double that of controls without diabetes. However, the overall costs of treating diabetes in 2022 appear to be stable compared with 2013. Expanding access to new treatments and technologies could help improve clinical outcomes in T2D, potentially leading to reductions in costly complications. There is a need for improved health policies for people living with T2D in the overseas departments.

## 1 INTRODUCTION

The prevalence of diabetes is high and rising, with 589 million adults worldwide (11.1% of the global adult population) estimated to have the condition in 2024.^1^ The characteristics of the population with diabetes are also changing.^1^ Over time, there has been a trend toward earlier, more effective treatment for patients with diabetes, with increasingly aggressive treatment targets and greater use of drugs that can protect organs from the damage associated with diabetes.^2^ This has led to people with diabetes living longer, often with chronic illnesses (not necessarily related to diabetes),^1^, ^2^ which has implications for management and outcomes.

In France, official epidemiological data suggest that 3.8 million individuals (5.6% of the overall French population including main overseas territories) were treated pharmacologically for diabetes in 2023.^3^ The corresponding figure in 2012 was 4.6% and there is some recent evidence that type 2 diabetes (T2D) prevalence is plateauing while type 1 diabetes (T1D) prevalence in people <20 years old is increasing (from 20 300 young people in 2012 to 31 400 in 2023).^3^

Most cases of diabetes in France, as in other countries, correspond to T2D. In 2019, 94% of identified patients with diabetes were determined to have T2D,^4^ although the genetic complexity of diabetes, for example, when individuals have maturity‐onset diabetes of the young (MODY; a form of monogenic diabetes),^5^ makes the true prevalence of T2D somewhat uncertain.

The large number of individuals living with T2D in France, together with the multiple complications that can be associated with the disease, results in a substantial economic burden. The last publications exploring the burden of T2D in France were published in 2018, using data from 2012^6^ or 2013.^7^ In the study using 2013 data, Charbonnel et al. compared French national health insurance data for 25 987 patients with T2D with a control group of 76 406 individuals without diabetes.^7^ Overall medical expenditure, per patient per year, was 6506€ in the T2D group and 3668€ in the control group. The 2838€ difference was mainly due to costs associated with hospitalization, medication, and nursing care. At the level of the overall population, the total direct cost of diagnosed T2D in France was estimated to exceed 8.5 billion €.^7^

T2D management has evolved over the last two decades. The first glucagon‐like peptide 1 receptor agonist (GLP‐1 RA) therapy was approved in Europe in 2006 and was available in France in 2007^7^; multiple agents in this class are now available. In addition, sodium‐glucose cotransporter 2 inhibitor (SGLT2i) treatment has been available for patients with T2D in France since 2020.^8^ Scientific guidelines have recently been updated, considering new alternatives and positive outcomes on microvascular and macrovascular complications, and positioning SGLT2i and GLP‐1 RA as recommended agents for T2D patients with comorbidities, with their use also increasing early in the T2D treatment pathway to reduce the risk of developing complications.^9^, ^10^, ^11^

The introduction of sensor‐based continuous glucose monitoring (CGM) technologies has also transformed diabetes management, providing patients with alternatives to capillary‐based self‐monitoring of blood glucose (SMBG). For example, in France FreeStyle Libre systems have been reimbursed since 2017 (and other sensor systems more recently) for glucose monitoring for patients with T1D and T2D who are using multiple daily injections of insulin (MDI).

In the context of these recent advances in T2D management, we sought to take a similar approach to the one previously described by Charbonnel et al.,^7^ in order to provide a comprehensive update of the treatment, medication patterns, and economic burden of T2D in France. The primary objectives of this study were: to describe the characteristics of patients with T2D; to describe the current management of T2D, including use of glucose‐lowering medications; to estimate the healthcare resource utilization and costs for a cohort of patients with T2D, compared with a control group without diabetes; and to estimate medical costs according to patients' treatment regimens.

## 2 METHODS

### 2.1 Study design

This was a descriptive retrospective cross‐sectional study of a representative sample of adult patients with T2D identified in the French national healthcare system claims database. Data for the year 2022 were the latest available in the database. Costs for patients with T2D were compared with a matched cohort without diabetes.

### 2.2 Data source

The Système National des Données de Santé (SNDS) is the national reimbursement claim database, which includes data for approximately 95% of the French population, including health insurance data with reimbursement claims for any ambulatory care consumption including medication and medical devices, acute care, public and private hospital data including ICD‐10 codes for primary, associated, and related diagnostics (DP, DR, DAS respectively), and disability‐related data. Because of the high prevalence of diabetes in France, the Echantillon Système National des Données de santé (ESND) database, which comprises a random sample of 2% of patients in the SNDS, was used, as recommended by the Comité d'Éthique et Scientifique pour les Recherches, les études et Évaluations dans le domaine de Santé.

### 2.3 Identification of adult patients with T2D

Adult patients with diabetes in 2021 or 2022 were identified as described in Charbonnel et al., 2018,^7^ based on: actual reimbursements for hypoglycemic drugs (European Pharmaceutical Market Research Association class A10; at least three reimbursements for hypoglycemic drugs, or two in the case of large packs, on separate dates in 2021 or 2022); a code for diabetes (affection de longue durée [ALD] 8) among the list of long‐term diseases from the National Sickness Fund; or a code related to diabetes as a main or secondary diagnosis in any hospital stay (Table S1).^12^

Patients with T2D were distinguished from those with T1D using the classification algorithm employed by Charbonnel et al.^7^, ^13^ This assumes that patients with T1DM will have had an ALD status or been hospitalized with a relevant International Classification of Diseases 10th Revision (ICD‐10) code over a 2‐year period (ICD‐10 codes: T1D, E10; T2D, E11). Diagnosis of MODY, which relies on genetic analysis, is typically conducted in hospitals and could not be assessed with the current algorithm.^5^, ^14^

Since the Charbonnel et al. analysis, SGLT2i therapies have been granted reimbursement in new indications outside T2D (chronic heart failure and chronic renal failure). In addition, GLP‐1 RA therapies may be used for weight loss by patients without diabetes. Accordingly, patients identified only through reimbursement of SGLT2i or GLP‐1 RA therapies (i.e., without any prescription of another antidiabetic drug; only 2% of prescriptions were in this category) were excluded.

Among the identified patients with T2D, only adults (aged 18 years and older) who were alive on 31 December 2022 were included. In those patients a look‐back window over the period 2018–2022 was used to identify comorbidities and complications. Patients newly diagnosed with T2D during this retrospective period were included in the analysis. Patients who were pregnant in 2022 or who were identified only through the hospitalization criterion were also excluded.

### 2.4 Identification of controls

A control group without diabetes was selected randomly in the ESND database. The control sample was matched using a propensity score based on: age (5‐year age groups); sex (male/female); geographical area (13 French regions); health coverage (Complémentaire Santé Solidaire [C2S; supplementary health insurance for deprived people] or Aide Médicale de l'État [AME; state medical assistance]); social deprivation index (quintile); and the presence of cancer unrelated to diabetes between 2017 and 2021 (all cancers except colorectal, hepatocellular, gallbladder, breast, endometrial, and pancreatic cancers). Where possible, a ratio of three controls to each case was used to maximize statistical power, as recommended by Woodward.^15^

### 2.5 Data collected and cost estimation

Data for eligible patients and controls were extracted from the database and included demographic information, treatment data, comorbidities, and complications.

Analysis of direct all‐cause healthcare expenditure (2022 euros) was carried out from an ‘extended’ health insurance perspective as it included reimbursed costs as well as co‐payments. Individuals presenting with severe chronic diseases belonging to a pre‐defined list of around 30 diseases (including diabetes mellitus) are eligible for full coverage (no co‐payment) of their disease‐related healthcare expenditures. This ALD status is documented by ICD‐10 codes. This information is currently used to document severe chronic comorbidities.

Ambulatory care costs (including medication deliveries) were taken directly from the reimbursement data in the database and correspond to the amounts of claims on the date of care. Hospital care costs were estimated according to their respective DRGs.

The economic burden of T2D was assessed by comparing the cost of all‐cause healthcare expenditures in the T2D population with that of controls. This difference represents the specific attributable direct economic cost associated with T2D.

### 2.6 Statistical analysis

Data for cases and controls were compared using the doubly robust augmented inverse probability weighting by propensity score method.^16^, ^17^ Outcome regression and propensity score matching methods can potentially be subject to bias if the statistical model is incorrectly specified. The doubly robust approaches combine the two methods, meaning that only one of the two models needs to be correctly specified in order for the effect estimator to be unbiased.^16^

All analyses were conducted using SAS software v9.4 (Cary, North Carolina). The covariates used were: age in 2022 (categories), sex, region, C2S, AME, deprivation index (quintile), comorbidities, and complications (20 variables).

Data were analysed for the overall population, and for the subgroups of patients living in metropolitan France and in French overseas departments (Guadeloupe, Martinique, Guyane, and La Réunion).

## 3 RESULTS

### 3.1 Study population

**FIGURE 1:**
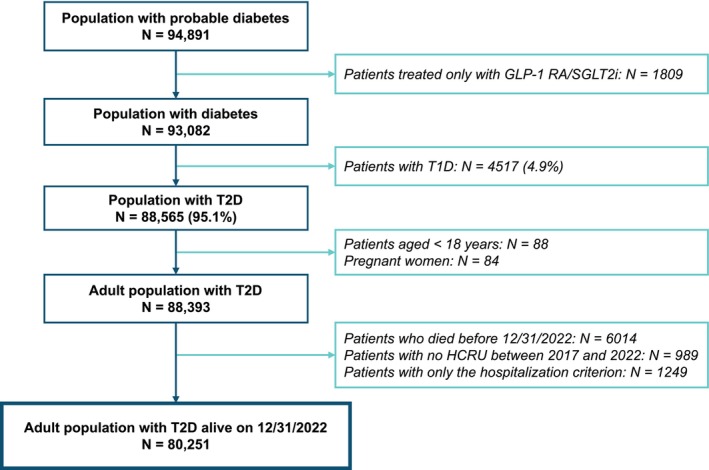
Identification of patients with T2D. Exclusion criteria are not mutually exclusive. GLP‐1 RA, glucagon‐like peptide 1 receptor agonist; HCRU, healthcare resource utilization; SGLT2i, sodium‐glucose cotransporter 2 inhibitor; T1D, type 1 diabetes; T2D, type 2 diabetes.

In total, 94 891 patients with diabetes were identified in the ESND database (Figure 1). After exclusion of patients with T1D, those aged <18 years, pregnant women, patients who died before the end of 2022, patients without recorded healthcare resource utilization between 2017 and 2022, and those only with codes related to hospitalization, 80 251 patients with T2D were identified.

After propensity score matching, 80 127 patients were matched to 237 607 controls: 92.4% of patients were matched with three controls; due to the rarity of some combinations of variables in the database, 6.9% could be matched with only two controls, and 0.6% with only one. A total of 124 patients with T2D could not be matched with a control; compared with the matched population, these patients were typically older, and almost all were in receipt of C2S (Table S2).

Overall, the mean age of patients with T2D was 68.4 years, and 54.5% were men (Table 1). Population characteristics were generally similar to those reported for 2013 by Charbonnel et al. (mean age, 67.2 years; % men, 54.1%).^7^

**TABLE 1:** Study population demographics.

Characteristics	Cases (*N* = 80 127)	Controls (*N* = 237 607)	Metropolitan France (*N* = 76213)	Overseas departments (*N* = 3608)	*p* value^a^
Age (years), mean ± SD	68.4 ± 13.0	68.0 ± 13.2	68.5 ± 12.9	64.8 ± 13.2	<0.0001
Median (range)	69 (18–109)	69 (18–110)	70 (18–109)	65 (18–100)	
Under 40 years (%)	1978 (2.5%)	6274 (2.6%)	1816 (2.4%)	146 (4.0%)	
40–49 years (%)	4367 (5.5%)	13341 (5.6%)	4075 (5.3%)	264 (7.3%)	
50–59 years (%)	12217 (15.2%)	37410 (15.7%)	11387 (14.9%)	782 (21.7%)	
60–69 years (%)	21838 (27.3%)	65112 (27.4%)	20615 (27.0%)	1130 (31.3%)	
70–79 years (%)	24245 (30.3%)	69658 (29.3%)	23396 (30.7%)	811 (22.5%)	
80 years and over (%)	15482 (19.3%)	45812 (19.3%)	14924 (19.6%)	475 (13.2%)	
Sex					
Men (%)	43945 (54.8%)	128690 (54.2%)	42169 (55.3%)	1609 (44.6%)	<0.0001
C2S^b^					
Yes (%)	8950 (11.2%)	24516 (10.3%)	7770 (10.2%)	1263 (35.0%)	<0.0001
AME					
Yes (%)	102 (0.1%)	169 (0.1%)	96 (0.1%)	5 (0.1%)	0.8080
Deprivation index					0.2678
Missing data	6547	18966	2879	3527	
1 (most favoured)	11168 (15.2%)	33868 (15.5%)	11116 (15.2%)	18 (22.2%)	
2	13052 (17.7%)	39167 (17.9%)	13005 (17.7%)	14 (17.3%)	
3	14713 (20.0%)	44183 (20.2%)	14675 (20.0%)	11 (13.6%)	
4	16268 (22.1%)	48258 (22.1%)	16219 (22.1%)	15 (18.5%)	
5 (most disadvantaged)	18379 (25.0%)	53165 (24.3%)	18319 (25.0%)	23 (28.4%)	
Presence of a non‐diabetes‐related cancer between 2017 and 2021					
Yes (%)	7563 (9.4%)	22468 (9.5%)	–	–	

Abbreviations: AME, Aide médicale de l'État (State Medical Assistance); C2S, Complémentaire Santé Solidaire (complementary health solidarity); SD, standard deviation.

^a^ *p* value for metropolitan France vs. overseas departments.

^b^ C2S covers the part of health expenses not reimbursed by health insurance.

Compared with metropolitan France, patients in overseas departments were on average younger and more likely to be female and in receipt of C2S (Table 1).

### 3.2 Comorbidities and complications

The presence of comorbidities and complications is summarized in Table 2. In total, 86.0% of patients with T2D had an ALD 8 code, corresponding to diabetes, compared with 74.4% in 2013.^7^ Long‐term disease codes for myocardial infarction, severe arrhythmia/heart disease, psychiatric disorder, arteriopathy, hypertension, stroke, chronic respiratory failure, and chronic liver disease were statistically significantly more common among patients with T2D than among controls.

**TABLE 2:** Comorbidities and complications.

Comorbidity/complication	Cases (*N* = 80 127)	Controls (*N* = 237 607)	*RR*	*p* ^a^	Metropolitan France (*N* = 76213)	Overseas departments (*N* = 3608)	*p* ^b^
Top 10 long‐term diseases							
ALD 8: Type 1 diabetes and type 2 diabetes in adults or children	86.0%	0.0%	—	<0.0001			
ALD 13: Coronary artery disease: myocardial infarction	9.8%	5.6%	—	<0.0001			
ALD 30: Malignant neoplasm (cancer), malignant disorder of lymphatic or haematopoietic tissue (e.g., lymphoma)	9.0%	9.7%	—	<0.0001			
ALD 5: Severe heart failure, serious arrhythmia, severe valvular heart disease, severe congenital heart disease	6.3%	6.0%	—	<0.0001			
ALD 23: Long‐term psychiatric disorders (e.g., recurrent depression, bipolar disorder)	4.2%	3.2%	—	<0.0001			
ALD 3: Chronic arteriopathies with ischemic manifestations	3.3%	2.7%	—	<0.0001			
ALD 12: Severe arterial hypertension	3.3%	1.1%	—	<0.0001			
ALD 1: Disabling stroke	2.3%	2.1%	—	<0.0001			
ALD 14: Severe chronic respiratory failure (e.g., severe asthma)	1.6%	1.2%	—	<0.0001			
ALD 6: Active chronic liver disease (hepatitis B or C) and cirrhosis	0.9%	0.5%	—	<0.0001			
ALD 9: Severe forms of neurological and muscle disorders (including myopathy), severe epilepsy	0.8%	1.0%	—	0.0006			
Microvascular complications							
Renal failure (dialysis/renal transplant/renal transplant follow‐up/ALD/RF hospitalization)	2379 (3.0%)	2558 (1.1%)	2.76 [2.61–2.91]	<0.0001	2247 (2.9%)	126 (3.5%)	0.0601
Retinal laser treatment (DP/DR H280 Cataract)	483 (0.6%)	18 (0.0%)	79.57 [49.71–127.37]	<0.0001	362 (0.5%)	124 (3.4%)	<0.0001
Diabetic retinopathy (DP/DR H360)	77 (0.1%)^c^	—	—	—	67 (0.1%)	<11	0.0072
Diabetic nephropathy (DP/DR N083)	63 (0.1%)^c^	—	—	—	58 (0.1%)	<11	0.2096
Macrovascular complications							
Treated hypertension in 2022 (at least 3 deliveries; ATC C02, C03, C07, C09, C08G)	58131 (72.5%)	99691 (42.0%)	1.73 [1.72–1.74]	<0.0001	55507 (72.8%)	2502 (69.3%)	<0.0001
Treated dyslipidemia in 2022 (at least 3 deliveries)	45395 (56.7%)	54414 (22.9%)	2.47 [2.45–2.50]	<0.0001	43695 (57.3%)	1596 (44.2%)	<0.0001
Coronary revascularization without infarction (CCAM codes)	3040 (3.8%)	3556 (1.5%)	2.54 [2.42–2.66]	<0.0001	2972 (3.9%)	62 (1.7%)	<0.0001
Heart failure (DP I50 or [DP I110, I130, I132, I139, K761, J81 with a DR or DAS I50])	1986 (2.5%)	1855 (0.8%)	3.17 [2.98–3.38]	<0.0001	1928 (2.5%)	52 (1.4%)	<0.0001
Myocardial infarction (DP I21, I22, I23, I24)	1905 (2.4%)	2624 (1.1%)	2.15 [2.03–2.28]	<0.0001	1831 (2.4%)	62 (1.7%)	0.0083
Stroke (DP I63, I64, I65, I66)	1718 (2.1%)	2879 (1.2%)	1.77 [1.67–1.88]	<0.0001	1641 (2.2%)	72 (2.0%)	0.5232
Arteriopathy of the lower limbs (DP/DR I702, I739, I740, I743, I744, I745)	1469 (1.8%)	1586 (0.7%)	2.75 [2.56–2.95]	<0.0001	1928 (2.5%)	52 (1.4%)	<0.0001
Unstable angina pectoris (DP/DR I200)	752 (0.9%)	1037 (0.4%)	2.15 [1.96–2.36]	<0.0001	731 (1.0%)	14 (0.4%)	0.0005
Transient ischemic attack (DP/DR G45 except G454)	458 (0.6%)	1001 (0.4%)	1.36 [1.22–1.51]	<0.0001	443 (0.6%)	18 (0.5%)	0.5234
Lower limb amputation (CCAM codes)	432 (0.5%)	173 (0.1%)	7.40 [6.21–8.83]	<0.0001	443 (0.6%)	18 (0.5%)	0.5234
Other complications							
Sleep Apnea (DP/DR G473 or LPP)	7500 (9.4%)	8173 (3.4%)	2.72 [2.64–2.80]	<0.0001	7229 (9.5%)	253 (7.0%)	<0.0001
Bariatric surgery (CCAM and ICD‐10 codes)	477 (0.6%)	447 (0.2%)	3.16 [2.78–3.60]	<0.0001	461 (0.6%)	14 (0.4%)	0.0979

*Note*: Exact patient numbers <11 are not shown to avoid revealing potentially identifying information.

Abbreviations: ALD, affection de longue durée (long‐term disease); CCAM, Classification Commune des Actes Médicaux (Common Classification of Medical Acts); DAS, associated diagnostic code; DP, primary diagnostic code; DR, related diagnostic code; ICD‐10, International Classification of Diseases 10th Revision; LLP, liste des Produits et Prestations (list of medical devices); RR, relative risk.

^a^ *p* value for cases versus controls.

^b^ *p* value for metropolitan France vs. overseas departments.

^c^ *n* = 80251.

Most patients with T2D had at least one recorded complication in the last 5 years (83.4%, compared with 49.0% in the control group).

Across all complications, including those not requiring hospitalization, the most frequently recorded were hypertension and dyslipidaemia. All complications listed in Table 2, including both microvascular and macrovascular complications, were statistically significantly more common among patients with T2D than in the control group.

Patients in overseas departments were less likely than those in metropolitan France to have hypertension, dyslipidaemia, sleep apnoea, coronary revascularization, heart failure, myocardial infarction, or angina, but more likely to have renal failure or to have experienced retinal laser treatment, lower limb amputation, or ketoacidosis (Table 2).

Compared with the overall population of patients with T2D, patients treated with GLP‐1 RA or SGLT2i therapies were more likely to have had at least one complication in the last 5 years (GLP‐1 RA, 90.6%; SGLT2i, 93.2%; overall population, 83.4%).

For complications reported by Charbonnel et al. in 2013,^7^ the prevalence in the 2022 ESND analysis was generally similar, although in both the T2D and control groups fewer patients were being treated for high blood pressure in 2022 (Table S3).

### 3.3 All‐cause hospitalization

All‐cause hospitalization (i.e., including hospitalization not related to diabetes) in the previous 5 years (2017–2021) was more common among patients with T2D than in the control group (68.1% vs. 57.4%, *p* < 0.0001). The proportion of patients with at least one recurrent day hospitalization (e.g., for chemotherapy or radiotherapy; 4.2% vs. 3.1%), at least 1 day hospitalization (47.6% vs. 41.8%) or at least one overnight stay (49.2% vs. 35.0%) were all statistically significantly higher in the T2D group than in the control group (all *p* < 0.0001), as was the mean number of stays among patients who were hospitalized (2.6 vs. 2.1, *p* < 0.0001). Compared with the control group, hospitalizations for patients in the T2D group were less likely to be in private hospitals (33.3% vs. 37.2%; *p* < 0.0001) or to be day cases (without an overnight stay; 16.4% vs. 22.9%; *p* < 0.0001 across all severity levels; Table S4).

### 3.4 Treatment

Treatment use during the last quarter of 2022 is shown in Table 3. In total, 66 891 patients (83.3% of the overall T2D cohort) received at least one diabetes treatment. During this period, insulin‐containing regimens were prescribed to 20.3% of treated patients, mostly basal insulin (11.2%) or MDI (8.3%) without continuous subcutaneous insulin infusion (CSII); 0.5% of treated patients were using CSII.

**TABLE 3:** T2D treatment regimens in Q4 2022.

Treatment	Overall study population (*N* = 80251)	Metropolitan France (*N* = 76213)	Overseas departments (*N* = 3608)	*p* ^a^
Any documented treatment	66 891 (100%)	63635 (100%)	2992 (100%)	
Monotherapy	26749 (40.0%)	25677 (40.4%)	984 (32.9%)	<0.0001
Metformin	31.1%	31.4%	25.1%	
Sulphonylurea/glinide	4.7%	4.7%	4.2%	
SGLT2i	0.7%	0.7%	0.5%	
GLP‐1 RA	0.9%	0.9%	0.9%	
DPP4i	2.4%	2.4%	1.7%	
Other monotherapies	0.2%	0.2%	0.4%	
Dual therapy	16592 (24.8%)	15784 (24.8%)	735 (24.6%)	<0.0001
Metformin + Sulphonylurea/glinide	5.4%	5.4%	6.3%	
Metformin + DPP4i	10.9%	10.9%	9.7%	
Metformin + GLP‐1 RA	3.7%	3.7%	3.7%	
Metformin + SGLT2i	1.8%	1.9%	1.2%	
Sulphonylurea/glinide + DPP4i	1.5%	1.5%	1.5%	
Sulphonylurea/glinide + GLP‐1 RA	0.6%	0.6%	0.6%	
Sulphonylurea/glinide + SGLT2i	0.3%	0.3%	0.3%	
SGLT2i + GLP‐1 RA	0.1%	0.1%	0.4%	
Other dual therapies	0.4%	0.4%	0.9%	
Triple therapy	8726 (13.0%)	8254 (13%)	436 (14.6%)	<0.0001
Metformin + Sulphonylurea/glinide + DPP4i	7.0%	6.9%	8.7%	
Metformin + Sulphonylurea/glinide + GLP‐1 RA	2.8%	2.8%	2.2%	
Metformin + Sulphonylurea/glinide + SGLT2i	0.8%	0.8%	0.8%	
Other triple therapy	2.4%	2.4%	2.9%	
Other multi‐therapy, excluding insulin	1268 (1.9%)	1190 (1.9%)	73 (2.4%)	<0.0001
Insulin regimens^b^	13556 (20.3%)	12730 (20.0%)	764 (25.5%)	<0.0001
Basal Insulin ± GLM without CSII	11.2%	10.9%	18.0%	
MDI ± GLM without CSII	8.3%	8.3%	7.3%	
CSII	0.5%	0.6%	0.1%	
Other	0.2%	0.2%	0.1%	

Abbreviations: CSII, continuous subcutaneous insulin infusion; DPP4i, dipeptidyl peptidase‐4 inhibitor; GLM, glucose‐lowering medication; GLP‐1 RA, glucagon‐like peptide 1 receptor agonist; MDI, multiple daily injections of insulin; SGLT2i, sodium‐glucose cotransporter 2 inhibitor.

^a^ *p* value for metropolitan France vs. overseas departments.

^b^ Patients receiving insulin may also be on SGLT2i and/or GLP‐1 RA therapies.

A total of 40.0% of treated patients were receiving non‐insulin monotherapy, most commonly metformin (31.1%). A further 24.8% were receiving dual therapy, with metformin plus a dipeptidyl peptidase‐4 inhibitor (DPP4i) the most frequently prescribed combination (10.9%). Triple therapy was prescribed for 13.0% of patients, most commonly metformin plus a DPP4i plus a sulphonylurea or glinide (7.0%).

In total, 11397 patients (17.0% of the treated cohort) were receiving a GLP‐1 RA therapy. Of these, 25.2% were also on basal insulin, 12.0% on MDI, 21.9% on metformin (only), and 16.3% on metformin plus a sulphonylurea or glinide (Table S5). SGLT1i prescriptions were recorded for 6596 (9.9%) treated patients, of whom 18.2% were also on basal insulin, 11.8% on MDI, 18.7% on metformin (only), and 28.6% on other triple‐ or multi‐therapy combinations (Table S6).

The distribution of patients across treatment lines in 2022 was highly consistent with the results reported by Charbonnel et al. for 2013, almost 10 years earlier^7^: monotherapy, 40.0% in 2022 versus 41.2% in 2013; dual therapy, 24.8% versus 25.6%; triple therapy, 13.0% versus 12.7%; insulin, 20.3% versus 19.4%.

Compared with metropolitan France, patients in overseas departments were more likely to be using insulin (25.5% vs. 20.0% of patients receiving any treatment for diabetes), and less likely to be on monotherapy (32.9% vs. 40.4%; Table 3).

A total of 43478 (65.0%) of the 66 891 treated patients remained on the same treatment line (i.e., always on monotherapy or always on dual therapy, but potentially with changes within those categories) throughout the year 2022. The highest percentage of patients changing their treatment line was observed in the insulin‐treated population (16% changed treatment line, compared with 0.9% of those on non‐insulin monotherapy, 2.4% of those on dual therapy, and 2.7% of those on triple therapy). Similar proportions changing treatment line were seen in metropolitan France and the overseas departments.

### 3.5 Glucose monitoring

Prescriptions for SMBG test strips in 2022 among patients who remained on the same treatment line during the year are shown in Table S7. The proportion of patients receiving prescriptions for test strips increased with increasing treatment line (monotherapy, 16.9%; dual therapy, 30.3%; triple therapy, 39.4%; basal insulin only, 80.2%; basal insulin plus glucose‐lowering medication, 81.9%). Similar results were seen for metropolitan France and overseas cohorts.

The proportion of patients receiving SMBG test strips in every quarter of 2022 among those who did not change their treatment regimen at all during the year is shown in Table S8. Among patients on monotherapy, dual therapy, and triple therapy, the proportion receiving test strips in every quarter was 10.8%, 13.5%, and 16.5%, respectively. Among these patients, the mean number of strips per day was similar in all three groups (monotherapy, 2.17; dual therapy, 2.13; triple therapy, 2.18). Patients on insulin‐containing regimens were more likely than those not on insulin to have at least one delivery of strips in every quarter (45.1% of patients, among whom a mean of 2.80 strips per day were received).

In the overall cohort, a total of 3742 patients had at least one delivery of CGM in the last quarter of 2022 (5.6% of the 66 891 treated patients). Most prescriptions for CGM (94.0%) were for patients treated with insulin; of the remainder, 2.1% were on monotherapy, 2.2% on dual therapy, and 1.5% on triple therapy.

### 3.6 Economic burden

**FIGURE 2:**
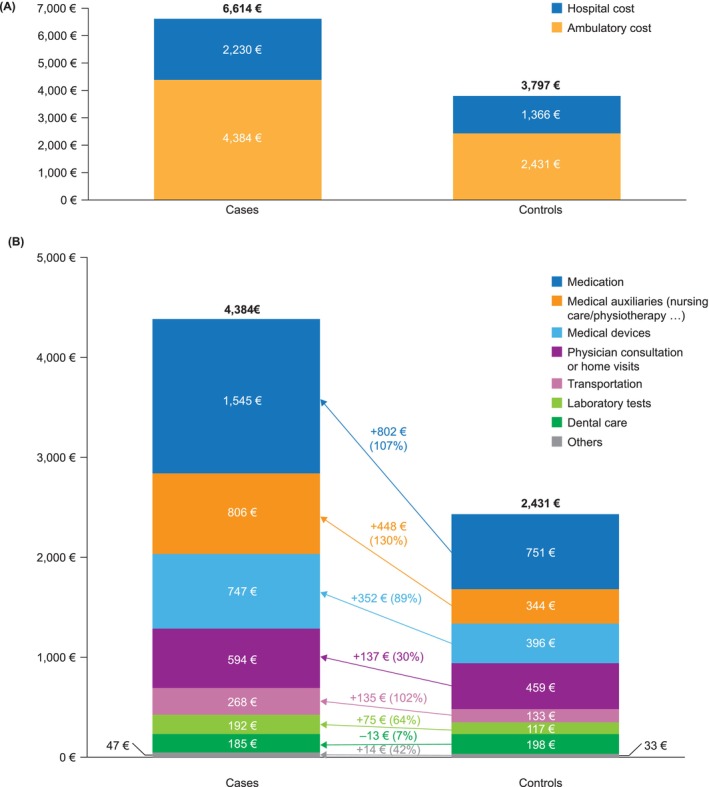
Annual costs for patients with diabetes and controls. *N* = 78820 T2D with at least one use of healthcare in 2022 versus 230 080 controls.

Annual costs for patients with at least one recorded use of healthcare in 2022 are shown in Figure 2. Overall, costs were higher for patients with T2D than for controls (6614€ [95% confidence interval {CI}, 6532–6696€] vs. 3797€ [95% CI, 3259–3834€]; difference, 2817€). The majority of costs in both groups were ambulatory (4384€ [95% CI, 4335–4431€] vs. 2431€ [95% CI, 2411–2450€]), with hospital costs comprising roughly a third of the total (2230€ [95% CI, 2175–2284€] vs. 1366€ [95% CI, 1337–1393€]). All cost components other than dental care were higher in the T2D group than in the control group, although there was only a small incremental cost of laboratory tests (75€).

In the adjusted model, using the doubly robust method, estimated costs were 6586€ [95% CI, 6505–6667€] in the T2D group and 3805€ [95% CI, 3767–3842€] in the control group (difference, 2781€).

Overall, T2D costs were higher for patients in overseas departments than for those in metropolitan France (Table S9). This difference was mainly due to an increased cost of medical auxiliaries (nursing care, etc., 1725€ vs. 750€ per patient per year).

Both total costs and the distribution of ambulatory and hospital costs in 2022 were similar to those reported by Charbonnel et al. for 2013 (2013 total costs: T2D group, 6506€; control group, 3668€).

Costs according to treatment regimen are shown in Table 4. Total costs, ambulatory costs and hospital costs were generally similar across non‐insulin monotherapy, dual therapy, and triple therapy regimens (total costs, 4878€, 4891€, and 4616€, respectively). Higher costs were seen among patients using basal insulin (total, 8554€) or MDI (total, 13913€). In particular, medical auxiliary care was more costly in the insulin‐using groups than among patients using non‐insulin therapies.

**TABLE 4:** Mean cost of T2D by treatment received (patients with the same treatment regimen throughout the year).

Cost	Glucose‐lowering medications												Insulin	
	All GLM *N* = 44671^a^	Monotherapy *N* = 19411	Dual therapy					Triple therapy					Basal insulin *N* = 4577	MDI/CSII *N* = 3852
			Total *N* = 10521	GLP‐1 RA *N* = 1560	SGLT2i *N* = 399	GLP‐1 RA plus SGLT2i *N* = 25	Other dual therapy *N* = 8537	Total *N* = 5273	GLP‐1 RA *N* = 1335	SGLT2 *N* = 302	GLP‐1 RA plus SGLT2i *N* = 100	Other triple therapy N = 3536		
**Ambulatory costs**	**3589 €**	**3392 €**	**3637 €**	**4639 €**	**4195 €**	**6535 €**	**3419€**	**3581 €**	**4392 €**	**4039 €**	**5107 €**	**3193 €**	**6599 €**	**10143 €**
Medication	1393 €	1172 €	1473 €	2131 €	1869 €	3998 €	1327 €	1596 €	2220 €	1830 €	2743 €	1308 €	2605 €	3229 €
Medical auxiliaries (nursing care, physiotherapy, etc.)	463 €	502 €	444 €	493 €	412 €	315 €	437€	369 €	415 €	398 €	432 €	347 €	1571 €	2391 €
Medical devices	559 €	550 €	561 €	730 €	617 €	700 €	528 €	521 €	625 €	501 €	710 €	478 €	1043 €	2573 €
Physician consultations or home visits	587 €	575 €	582 €	660 €	681 €	578 €	564 €	559 €	631 €	615 €	581 €	526 €	645 €	741 €
Transportation	172 €	186 €	163 €	188 €	172 €	383 €	158 €	143 €	118 €	138 €	136 €	153 €	303 €	666 €
Dental care	197 €	195 €	201 €	208 €	158 €	201 €	202 €	200 €	180 €	307 €	238 €	1928 €	172 €	199 €
Laboratory tests	181 €	177 €	174 €	186 €	234 €	294 €	169 €	163 €	169 €	207 €	215 €	155 €	205 €	265 €
Others	37 €	35 €	36 €	43 €	53 €	67 €	34 €	31 €	35 €	43 €	53 €	28 €	55 €	79 €
**Hospital costs**	**1464 €**	**1486 €**	**1255 €**	**1249 €**	**1983 €**	**4116 €**	**1213 €**	**1035 €**	**1155 €**	**1552 €**	**1215 €**	**941 €**	**1955 €**	**3770 €**
***Total 2022 Costs***	***5053 €***	***4878 €***	***4891 €***	***5888 €***	***6178 €***	***10651 €***	***4632 €***	***4616 €***	***5547 €***	***5591 €***	***6321 €***	***4134 €***	***8554 €***	***13913 €***
***95% CI of total costs***	***[4972–5135]***	***[4741–5014]***	***[4744–5038]***	***[5518–6258]***	***[5504–6851]***	***[6812–14490]***	***[4467–4796]***	***[4426–4807]***	***[5182–5811]***	***[4801–6379]***	***[5286–7356]***	***[3898–4369]***	***[8175–8932]***	***[13394–14432]***

*Note*: Patients receiving other multiple therapy regimens excluding insulin are not included in the analysis (*N* = 505). Bold indicates the specific ambulatory and hospital care costs according to the different profiles of patients with type 2 diabetes.

Abbreviations: CSII, continuous subcutaneous insulin infusion; GLM, glucose‐lowering medication; GLP‐1 RA, glucagon‐like peptide 1 receptor agonist; MDI, multiple daily injections of insulin; SGLT2i, sodium‐glucose cotransporter 2 inhibitor.

^a^ ‘All GLM’ column includes all patients who were on GLM throughout 2022 (but could have switched between monotherapy and dual therapy, etc.); other columns include only patients who remained on each treatment line (e.g., only monotherapy; only GLP‐1 RA dual therapy, etc.) throughout 2022.

## 4 DISCUSSION

This retrospective claims database study analysed the characteristics of the population of people living with T2D in France, their treatment, and the associated costs. For the year 2022, the incremental direct costs associated with T2D were €2817 per patient (6586€ vs. 3805€). Extrapolating to the overall population of people with T2D, the attributable direct cost of diagnosed T2D in France in 2022 can be estimated to be 11.2 billion € (2800€ × 50 × 80000).

This analysis updates the assessment of the treatment, medication patterns and economic burden of T2D in France in 2013 published by Charbonnel et al.^7^ A similar approach was taken, with the ESND sample of 2% of beneficiaries in the SNDS used in place of the older 1% Echantillon Généraliste des Béneficiaires sample used in the earlier study; as both are random samples from the same data source the two analyses should be comparable. Overall, the findings of the two studies are highly consistent, despite being almost 10 years apart. In addition to similar population characteristics and comorbidity profiles, the two analyses found a very similar distribution of patients across treatment regimens. Direct costs per patient per year were also similar in 2013 and 2022. The similarity of these findings is notable, given that changes in the age profile of the T2D population^1^ may have been expected to lead to changes in treatment and healthcare resource utilization. Broadly similar findings were seen in a recent UK study, which found that direct medical costs due to diabetes increased from £9.8 billion in 2012 to £10.7 billion in 2022 (without inflation adjustment).^18^, ^19^ By contrast, the total medical costs associated with diabetes in the USA have been estimated to have risen by 35% in real terms over the same period.^20^

Given the changes in various guidelines since 2013, it was of interest to investigate how the treatment of T2D in 2022 compared with the earlier study. It is notable that in 2022 the most common monotherapy, dual therapy, and triple therapy treatments were the same as in the 2013 study (metformin, 31.1% in 2022 vs. 27.3% in 2013; metformin plus DPP4i, 10.9% vs. 10.0%; and metformin plus DPP4i plus sulphonylurea/glinide, 7.0% vs. 8.4%). This suggests that clinical practice has been generally conservative between 2013 and 2022, with relatively little change in prescribing patterns. Overall, 83.3% of patients were receiving at least one diabetes treatment, consistent with recent figures from other European countries.^21^, ^22^

SGLT2i and GLP‐1 RA therapies were used by a relatively small proportion of patients in the 2022 data, particularly among patients not using insulin. This would be consistent with 2022 clinical practice having been largely based on Haute Autorité de Santé (HAS) guidelines from 2013^23^; it is therefore possible that prescribing patterns may change following the updated guidelines published by HAS and by the Société Francophone du Diabète in 2024 and 2023, respectively,^24^, ^25^ both of which recommend use of SGLT2i and GLP‐1 RA therapies earlier in the T2D treatment pathway. An additional factor may also have been recent shortages of GLP‐1 RA therapies.^26^ A higher proportion of SGLT2i use was seen in a recent German study, in which 29.1% of prescriptions for T2D in the first quarter of 2025 were for an SGLT2i and 12.9% were for a GLP‐1 RA/tirzepatide, a substantial increase from 2017 (SGLT2i, +18.4%; GLP‐1 RA/tirzepatide, +6.6%).^27^ In the Netherlands, SGLT‐2i therapies were prescribed more often than GLP‐1 RAs in 2022 (14.5% vs. 6.7%), with substantial increases noted versus 2021 and earlier years.^28^

Within the year 2022, patients' treatment regimens were mostly stable, with only 35% of patients receiving treatment for T2D changing their treatment during the year. Whereas 16% of patients using insulin changed their treatment class during the year, fewer than 1% of patients on non‐insulin monotherapy did so. This suggests that there may be a degree of treatment inertia, particularly in early treatment lines; this would be consistent with the relatively small increment in laboratory costs seen in the T2D group, compared with the control group. Treatment inertia in T2D is common and frequently reflects concerns about hypoglycaemia; however, it can mean that patients are not receiving optimal treatment and can lead to long‐term complications.^29^ However, in the absence of data on HbA1c levels, body mass index (BMI) or the duration of diabetes, it is impossible to distinguish appropriate stability from inertia. Nevertheless, these results suggest that expanding access to new treatments and glucose monitoring technologies could help improve clinical outcomes.

It is notable that while the proportion of the French population who are overweight or obese has increased in recent years,^30^ data from the third edition of the Echantillon National Témoin Représentatif des personnes Diabétiques (ENTRED 3) study show that the mean BMI of patients with T2D has remained relatively stable.^4^ It might be speculated that for a subset of patients with T2D, use of GLP‐1 RA therapies is contributing to a degree of control over obesity levels; it is also possible that this reflects patients modifying their diet and exercise in response to recommendations from their healthcare providers.

Charbonnel et al. found that the costs of T2D per patient per year had increased by 30% between 2007 and 2013.^7^ By contrast, costs were essentially stable in euro terms between 2013 and 2022; the effect of inflation over this period suggests that in real terms the costs of treating diabetes on a per‐patient, per‐year basis actually decreased. It is notable that even though newer treatments—SGLT2i and GLP‐1 RA therapies—are more costly than older agents such as metformin, the low level of penetration of these therapies means that the overall economic burden of diabetes has remained stable over almost 10 years. Although we hypothesized that the increased cost of newer treatments might be offset by reduced hospitalization costs due to their greater effectiveness, alongside a trend toward more treatment in outpatient settings and decreases in the number of hospital beds, the incremental hospitalization costs in the 2022 and 2013 T2D cohorts, compared with the corresponding control groups, were similar (864€ vs. 855€). These findings therefore appear to reflect the effectiveness of the French national pricing policy, although multiple alternative explanations may also contribute, including changes in case‐mix, shifting of care from inpatient to outpatient settings, coding changes, or under‐penetration of newer therapies.

It is unclear whether these effective cost‐control measures may have affected the penetrance of newer therapies. Since the prevalence of comorbidities and diabetes complications also appears to be generally similar in the 2022 and 2013 analyses, it is possible that at a population level the benefits of newer therapies for T2D are not being fully realized. Accordingly, while French reimbursement policies have successfully managed the costs of treating diabetes, this may not have resulted in the optimal compromise between efficiency and patient outcomes.

As in the Charbonnel et al. study, the largest individual cost components associated with T2D were hospitalization, medication, and auxiliary care (nursing care and physiotherapy). As noted by Charbonnel et al.,^7^ some patients with T2D in France may be receiving treatment in hospital for issues that in other healthcare systems might be treated in another setting. It is also apparent that, as in 2013,^7^ patients with T2D, and particularly those on insulin, are in receipt of a considerable level of nursing care.

In total, 5.6% of patients receiving diabetes treatment received a CGM prescription in the last quarter of 2022. The data used in this analysis predate the French approval in 2023 of FreeStyle Libre 2 for people with T2D using basal insulin. This may lead to greater use of CGM in future among this patient group, for whom the benefits of the technology have been demonstrated in previous studies in multiple countries, including France.^31^, ^32^, ^33^, ^34^, ^35^

The results of this study have shown that the use of new treatments for T2D is still relatively limited in France. It remains unclear whether this reflects pricing policies, treatment inertia, or both. However, the use of GLP‐1 RA and/or SGLT2i therapies by people with T2D has been found to statistically significantly reduce the risk of myocardial infarction, heart failure, and stroke, and to improve kidney outcomes.^36^, ^37^, ^38^, ^39^, ^40^ Therefore, the higher prevalence of complications seen in patients receiving these therapies is consistent with their use to improve outcomes in patients with these conditions. As for CGM technology, broadening access to newer treatment options may improve clinical outcomes and, by reducing the incidence of costly complications, could help reduce the economic burden of diabetes.

The ENDS database includes data for patients living in four French overseas departments—Guadeloupe, Martinique, Guyane, and La Réunion—which have a higher prevalence of diabetes than metropolitan France.^41^ As reported in ENTRED 3,^42^ patients in overseas departments were on average younger and more likely to be female, differences which may explain the different complication profiles seen in the two populations. Despite their lower mean age, patients in the overseas territories were more likely than those in metropolitan France to be using insulin, a difference also seen in ENTRED 3.^42^ Auxiliary care (nursing care, etc., 1725€ vs. 750€ per patient per year) was the main driver of the higher cost of T2D in the overseas departments, compared with metropolitan France. These findings suggest that, in addition to differences in age, type of diabetes, and ethnic diversity compared with metropolitan France, issues may exist in the overseas departments around health and diabetes management education and the appropriate use of treatments for insulin resistance.

The lower prevalence of some complications in the overseas territories might indicate that these complications are detected less reliably than in metropolitan France. However, while the ENTRED 3 study found lower rates of dyslipidaemia in multiple overseas territories, compared with metropolitan France, a lower rate of hypertension was seen only in Guyana.^42^ The possibility of issues concerning the recording of complications means that caution is needed in these comparisons. Overall, these results suggest that there is an urgent need for a tailored health policy for individuals living with T2D in the overseas territories.

### 4.1 Limitations

This study has some limitations. First, the SNDS database does not include test results, and therefore HbA1c data could not be assessed. Second, identification of patients with T2D relied mainly on excluding those with T1D, which may not be completely accurate, with the presence in real‐world practice of complex cases and conditions such as MODY potentially complicating the identification of patients with T2D in the database. However, the predominance of T2D means that these issues are likely to have only a small impact on the results. Third, the real‐world data used in this analysis include several potentially important sources of bias, including potential misclassification of complications, lack of data on diabetes duration and lifestyle factors, and residual confounding between T2D and controls despite matching. Fourth, acute diabetes events (ketoacidosis, hyperglycaemia, hypoglycaemia, and coma) were reported in the database only at a very low frequency, suggesting substantial under‐ascertainment. These events were therefore not included in the analysis. Fifth, the results could not be broken down by sex. Sixth, the analysis was conducted using data from 2022 and does not reflect the approval of FreeStyle Libre 2 for T2D treated with basal insulin or the 2024 update to the HAS guidelines for the treatment of T2D.

## 5 CONCLUSIONS

The total direct costs of T2D in 2022 in France were estimated to be 11.3 billion €, indicating that T2D continues to represent a significant economic burden. The costs of treating diabetes in 2022 appear to be stable in comparison with 2013. However, while the number of new therapies and glucose monitoring devices has increased since 2013, prescribing patterns are generally consistent, with a relatively low uptake of new technologies and indications of treatment inertia. The prevalence of diabetes complications does not appear to have decreased since 2013, suggesting that there is still scope for improvements in clinical management. There is also a pressing need to address the aging of the T2D population and the challenges this presents for appropriate care. Expanding access to new treatments and technologies, although more costly than older alternatives, could help improve clinical outcomes in T2D, potentially leading to a reduction in costly complications. These findings also identify a need for improved health policies for people living with T2D in the overseas departments.

## AUTHOR CONTRIBUTIONS

**Bruno Guerci**: Study design and manuscript preparation. **Christèle Blanc‐Bisson**: Study design and manuscript preparation. **Eric Vicaut**: Study design and manuscript preparation. **Gérard De Pouvourville**: Study design and manuscript preparation. **Bruno Detournay**: Study design and manuscript preparation. **Corinne Emery**: Data extraction, statistical analysis, and manuscript preparation. **Isabelle Bureau**: Data extraction, statistical analysis, and manuscript preparation. **Taos Ihaddadene‐Salzgeber**: Study design and manuscript preparation. **Fleur Levrat‐Guillen**: Study design and manuscript preparation. **Jean‐Pierre Riveline**: Study design and manuscript preparation. Bruno Guerci is the guarantor of this work and, as such, had full access to all the data in the study and takes responsibility for the integrity of the data and the accuracy of the data analysis.

## CONFLICT OF INTEREST STATEMENT

The authors acknowledge funding support from Abbott Diabetes Care. The funding did not affect the collection, analysis, or presentation of the data.

## Supporting information


**Table S1.** Codes used to identify diabetes cases.
**Table S2.** Comparison of patients with T2D who were and were not matched with controls.
**Table S3.** Comparison of comorbidities and diabetes complications between 2022 ESND cohort and 2013 Charbonnel et al. analysis.
**Table S4.** Comparison of hospitalization severity levels between cases and controls.
**Table S5.** T2D treatment regimens in Q4 2022 among patients receiving GLP‐1 RA therapy.
**Table S6.** T2D treatment regimens in Q4 2022 among patients receiving SGLT2i therapy.
**Table S7.** Prescription of SMBG test strips in 2022 among patients who remained on the same treatment line during the year.
**Table S8.** Prescription of SMBG test strips in every quarter of 2022, by treatment regimen, among patients receiving the same treatment throughout 2022 and with at least one delivery strips in 2022.
**Table S9.** Comparison of annual costs between metropolitan France and overseas departments.

## Data Availability

The data in this study were obtained from the French nationwide claims and hospitalization database, the SNDS, access to which is regulated by the Commission Nationale de l'Informatique et des Libertés.
