# Supplementary material for: Economic burden of type 2 diabetes management in France according to clinical characteristics
Source: Diabetes Obes Metab. 2026 Mar 9;28(5):4019–32. doi: 10.1111/dom.70586 (PMC13071239; doi:10.1111/dom.70586)
Supplement: Supplementary file 1 — Table S1. Codes used to identify diabetes cases. Table S2. Comparison of patients with T2D who were and were not matched with controls. Table S3. Comparison of comorbidities and diabetes complications between 2022 ESND cohort and 2013 Charbonnel et al. analysis. Table S4. Comparison of hospitalization severity levels between cases and controls. Table S5. T2D treatment regimens in Q4 2022 among patients receiving GLP‐1 RA therapy. Table S6. T2D treatment regimens in Q4 2022 among patients receiving SGLT2i therapy. Table S7. Prescription of SMBG test strips in 2022 among patients who remained on the same treatment line during the year. Table S8. Prescription of SMBG test strips in every quarter of 2022, by treatment regimen, among patients receiving the same treatment throughout 2022 and with at least one delivery strips in 2022. Table S9. Comparison of annual costs between metropolitan France and overseas departments. [file DOM-28-4019-s001.docx]

# Supplementary material

# Economic impact of type 2 diabetes management in France according to clinical characteristics

*Bruno Guerci ^a^, Christèle Blanc-Bisson ^b^, Eric Vicaut ^c^, Gérard De Pouvourville ^d^, Bruno Detournay ^e^, Corinne Emery ^e^, Isabelle Bureau ^e^, Taos Ihaddadene-Salzgeber ^f^, Fleur Levrat-Guillen ^g^, Jean-Pierre Riveline ^h i^*

*a.* *University of Lorraine, CHRU Nancy, Brabois Hospitals, Department of Endocrinology-Diabetology-Nutrition, Vandoeuvre-les-Nancy, France*

*b. Département de Médecine Générale, Bordeaux University, Bordeaux, France*

*c. URC HUSLS, Lariboisière Fernand Vidal Hospital, Paris, France*

*d. Department of Economics, ESSEC Business School, Paris France*

*e. CEMKA, Bourg-La-Reine, France*

*f. Abbott France, Rungis, France*

*g. Abbott Ltd, Maidenhead, UK*

*h. Department of Diabetology and Endocrinology, Lariboisiere Hospital, Assistance Publique - Hopitaux de Paris, Paris, France*

*i. Unite INSERM U1138 Immunity and Metabolism in Diabetes, ImMeDiab Team Institut Necker Enfants Malades and Université de Paris Cité, Paris, France.*

# Corresponding author: Bruno Guerci

Full postal address: University of Lorraine, CHRU Nancy, Brabois Hospitals, Department of Endocrinology-Diabetology-Nutrition, Vandoeuvre-les-Nancy, France

Email: b.guerci@chru-nancy.fr

Supplementary Table 1 Codes used to identify diabetes cases

| **ICD-10 code** | **Term** |
| --- | --- |
| *Hospital database and ALD (diagnostics)* | |
| E10 | Diabète sucré insulino-dépendant (insulin-dependent diabetes mellitus) |
| E11 | Diabète sucré non insulino-dépendant (non-insulin-dependent diabetes mellitus) |
| E12 | Diabète sucré de malnutrition (malnutritional diabetes mellitus) |
| E13 | Autres diabètes sucrés précisés (other specified diabetes mellitus) |
| E14 | Diabète sucré, sans précision (unspecified diabetes mellitus) |
| *Hospital database (complications of diabetes)* | |
| G59.0* | Mononévrite diabétique (diabetic mononeuritis) |
| G63.2* | Polynévrite diabétique (diabetic polyneuritis) |
| G73.0* | Syndrome myasthénique au cours de maladie endocrinienne (myasthenic syndrome in endocrine disease) |
| G99.0* | Neuropathie du système nerveux autonome au cours maladies endocriniennes et métaboliques (autonomic neuropathy in endocrine and metabolic diseases) |
| H28.0* | Cataracte diabétique (diabetic cataract) |
| H36.0* | Rétinopathie diabétique (diabetic retinopathy) |
| I79.2* | Angiopathie périphérique au cours de maladies classées ailleurs (peripheral angiopathy in diseases classified elsewhere) |
| L97 | ulcère du membre inférieur, non classé ailleurs (lower limb ulcer, not elsewhere classified) |
| M14.2* | Arthropathie diabétique (diabetic arthropathy) |
| M14.6* | Arthropathie nerveuse (nervous arthropathy) |

ICD-10, International Classification of Diseases 10th Revision.

Source: l’Assurance Maladie [16].

Supplementary Table 2 Comparison of patients with T2D who were and were not matched with controls

| **Characteristic** | **Cases with controls (N = 80,127)** | **Cases without controls (N = 124)** |
| --- | --- | --- |
| Age (years), mean+/-SD | 68.4 +/- 13.0 | 75.7 +/- 5.1 |
| Median (range) | 69 (18 – 109) | 76 (66 – 90) |
| Under 40 years (%) | 1978 (2.5%) |  |
| 40–49 years (%) | 4367 (5.5%) |  |
| 50–59 years (%) | 12,217 (15.2%) |  |
| 60–69 years (%) | 21,838 (27.3%) | 10 (8.1%) |
| 70–79 years (%) | 24,245 (30.3%) | 94 (75.8%) |
| 80 years and over (%) | 15,482 (19.3%) | 20 (16.1%) |
| Sex |  |  |
| Men (%) | 43,945 (54.8%) | 61 (49.2%) |
| C2S |  |  |
| Yes (%) | 8950 (11.2%) | 123 (99.2%) |
| AME |  |  |
| Yes (%) | 102 (0.1%) | 0 (0.0%) |
| Deprivation index |  |  |
| 1 (most favored) | 11,168 (15.2%) | 0 (0.0%) |
| 2 | 13,052 (17.7%) | 9 (18.0%) |
| 3 | 14,713 (20.0%) | 17 (34.0%) |
| 4 | 16,268 (22.1%) | 9 (18.0%) |
| 5 (most disadvantaged) | 18,379 (25.0%) | 15 (30.0%) |
| Missing data | 6547 | 74 |

AME, Aide médicale de l'État (State Medical Assistance); C2S, Complémentaire Santé Solidaire (complementary health solidarity; this aid covers the part of health expenses not reimbursed by health insurance); SD, standard deviation.

Supplementary Table 3 Comparison of comorbidities and diabetes complications between 2022 ESND cohort and 2013 Charbonnel et al. analysis

| **Comorbidities and complications** | **Cases** | **Controls** | ***p*** |
| --- | --- | --- | --- |
| ***ESND analysis (2017–2021)*** | ***N = 80,127*** | ***N = 237,607*** |  |
| Sleep Apnea Hospitalization (DP/DR G473) | 1608 (2.0%) | 2064 (0.9%) | < 0.0001 |
| Treated dyslipidemia in 2022 (at least 3 deliveries) | 45,395 (56.7%) | 54,414 (22.9%) | < 0.0001 |
| Treated hypertension in 2022 (at least 3 deliveries; ATC C02, C03, C07, C09, C08G) | 58,131 (72.5%) | 99,691 (42.0%) | < 0.0001 |
| Retinal laser treatment (DP/DR H280) | 483 (0.6%) | 18 (0.0%) | < 0.0001 |
| At least one cancer unrelated to diabetes (Hospitalization DP/DR/DAS MCO and/or LTD) (all cancers except colorectal, hepatocellular, gallbladder, breast, endometrial, and pancreatic cancers) | 7,563 (9.4%) | 22,468 (9.5%) | 0,8857 |
| Prostate (C61) | 2,228 (2.78%) | 7,068 (2.97%) |  |
| Bladder (C67) | 739 (0.92%) | 1986 (0.84%) |  |
| Lung (C34) | 491 (0.61%) | 1403 (0.59%) |  |
| ***Charbonnel et al. 2013 analysis*** | ***N = 25,987*** | ***N = 76,406*** |  |
| Sleep Apnea Hospitalization (DP/DR G473) | 2.2% | 0.6% | < 0.0001 |
| Treated dyslipidemia in 2013 | 59.4% | 28.6% | < 0.0001 |
| Treated hypertension in 2013 | 77.7% | 48.1% | < 0.0001 |
| Retinal laser treatment in 2013 | 0.3% | <0.1% | < 0.0001 |
| At least one cancer | 12.2% | 11.8% | 0.0615 |
| Prostate (C61) | 593 (2.3%) | 1964 (2.6%) | 0.0002 |
| Bladder (C67) | 206 (0.79%) | 521 (0.68%) | 0.1517 |
| Lung (C34) | 131 (0.50%) | 356 (0.46%) | 0.6112 |

Supplementary Table 4 Comparison of hospitalization severity levels between cases and controls

|  | **Cases** | **Controls** | **p value** |
| --- | --- | --- | --- |
| Severity level |  |  | < 0.0001 |
| 1 | 15.0% | 15.7% |  |
| 2 | 8.8% | 6.7% |  |
| 3 | 5.0% | 4.1% |  |
| 4 | 1.6% | 1.2% |  |
| J (day cases; LOS = 0) | 16.4% | 22.9% |  |
| T (short LOS) | 10.4% | 8.9% |  |
| Z (not concerned by a severity level) | 42.9% | 40.6% |  |

LOS, length of stay.

Supplementary Table 5 T2D treatment regimens in Q4 2022 among patients receiving GLP-1 RA therapy

|  | **Overall study population (N = 80,251)** | **Metropolitan France (N = 76,213)** | **Overseas departments (N = 3608)** | ***p*** |
| --- | --- | --- | --- | --- |
| Any documented treatment | 66,891 (100%) | 63,635 (100%) | 2992 (100%) |  |
| Patients receiving GLP1-RA | 11,397 (17.0%) | 10,800 (17.0%) | 549 (18.3%) | 0.0789 |
| If yes: |  |  |  |  |
| Monotherapy |  |  |  | < 0.0001 |
| GLP-1 RA | 607 (5.3%) | 576 (5.3%) | 28 (5.1%) |  |
| Dual therapy |  |  |  |  |
| Metformin + GLP-1 RA | 2496 (21.9%) | 2372 (22.0%) | 111 (20.2%) |  |
| Sulphonylurea /glinide + GLP-1 RA | 382 (3.4%) | 360 (3.3%) | 18 (3.3%) |  |
| SGLT2i + GLP-1 RA | 98 (0.9%) | 84 (0.8%) | 13 (2.4%) |  |
| Other dual therapies including GLP‑1 RA | 37 (0.3%) | 33 (0.3%) | < 11 |  |
| Triple therapy |  |  |  |  |
| Metformin + Sulphonylurea/glinide + GLP-1 RA | 1858 (16.3%) | 1786 (16.5%) | 65 (11.8%) |  |
| Other triple therapy including GLP‑1 RA | 821 (7.2%) | 789 (7.3%) | 31 (5.6%) |  |
| Other multi-therapy including GLP‑1 RA, excluding insulin | 745 (6.5%) | 709 (6.6%) | 32 (5.8%) |  |
| Insulin regimens including GLP-1 RA |  |  |  |  |
| Basal insulin +/- GLM without CSII | 2867 (25.2%) | 2676 (24.8%) | 182 (33.2%) |  |
| Multi-injection +/- GLM without CSII | 1369 (12.0%) | 1299 (12.0%) | 65 (11.8%) |  |
| CSII | 92 (0.8%) | 91 (0.8%) | < 11 |  |
| Other insulin regimens | 25 (0.2%) | 25 (0.2%) | – |  |

Exact patient numbers < 11 are not shown to avoid revealing potentially identifying information.

CSII, continuous subcutaneous insulin infusion; DPP4i, dipeptidyl peptidase-4 inhibitor; GLM, glucose-lowering medication; GLP-1 RA, glucagon-like peptide 1 receptor agonist; MDI, multiple daily injections of insulin; SGLT2i, sodium-glucose cotransporter 2 inhibitor.

Supplementary Table 6 T2D treatment regimens in Q4 2022 among patients receiving SGLT2i therapy

|  | **Overall study population (N = 80,251)** | **Metropolitan France (N = 76,213)** | **Overseas departments (N = 3608)** | ***p*** |
| --- | --- | --- | --- | --- |
| Any documented treatment | 66,891 (100%) | 63,635 (100%) | 2992 (100%) |  |
| Patients receiving SGLT2i | 6596 (9.9%) | 6267 (9.8%) | 293 (9.8%) | 0.8272 |
| If yes: |  |  |  |  |
| Monotherapy |  |  |  | < 0.0001 |
| SGLT2i | 444 (6.7%) | 426 (6.8%) | 16 (5.5%) |  |
| Dual therapy |  |  |  |  |
| Metformin + SGLT2i | 1235 (18.7%) | 1195 (19.1%) | 35 (11.9%) |  |
| Sulphonylurea /glinide + SGLT2i | 200 (3.0%) | 188 (3.0%) | < 11 |  |
| SGLT2i + GLP1 | 98 (1.5%) | 84 (1.3%) | 13 (4.4%) |  |
| Other dual therapies including GLP‑1 RA | 126 (1.9%) | 118 (1.9%) | < 11 |  |
| Triple therapy |  |  |  |  |
| Metformin + Sulphonylurea/glinide + SGLT2i | 563 (8.5%) | 539 (8.6%) | 24 (8.2%) |  |
| Other triple therapy including SGLT2i | 1071 (16.2%) | 1018 (16.2%) | 49 (16.7%) |  |
| Other multi-therapy including SGLT2i, excluding insulin | 815 (12.4%) | 768 (12.3%) | 42 (14.3%) |  |
| Insulin regimens including SGLT2i |  |  |  |  |
| Basal insulin +/- GLM without CSII | 1198 (18.2%) | 1115 (17.8%) | 76 (25.9%) |  |
| Multi-injection +/- GLM without CSII | 780 (11.8%) | 750 (12.0%) | 23 (7.8%) |  |
| CSII | 51 (0.8%) | 51 (0.8%) | – |  |
| Other insulin regimens | 15 (0.2%) | 15 (0.2%) | – |  |

Exact patient numbers < 11 are not shown to avoid revealing potentially identifying information.

CSII, continuous subcutaneous insulin infusion; DPP4i, dipeptidyl peptidase-4 inhibitor; GLM, glucose-lowering medication; GLP-1 RA, glucagon-like peptide 1 receptor agonist; MDI, multiple daily injections of insulin; SGLT2i, sodium-glucose cotransporter 2 inhibitor.

Supplementary Table 7 Prescription of SMBG test strips in 2022 among patients who remained on the same treatment line during the year

| **Treatment** | **Patients with at least one delivery of strips in 2022** | **If yes, mean number of strips delivered per day** |
| --- | --- | --- |
| Monotherapy (N = 19,411) | 3272 (16.9%) | 0.7 (+/- 0.6) |
| Dual therapy (N = 10,521) | 3183 (30.3%) | 0.8 (+/- 0.7) |
| Triple therapy (N = 5273) | 2075 (39.4%) | 0.9 (+/- 0.8) |
| Insulin, basal only (N = 393) | 315 (80.2%) | 1.8 (+/- 1.0) |
| Insulin, basal with GLM (N = 3991) | 3269 (81.9%) | 1.8 (+/- 1.1) |
| Sulphonylurea/glinides (N=16,552) | 7545 (45.6%) | 1.2 (+/- 1.1) |

Data are for patients who remained on the same treatment line (i.e., always on monotherapy or always on dual therapy, but potentially with changes within those categories) throughout the year 2022.

GLM, glucose-lowering medication; GLP-1 RA, glucagon-like peptide 1 receptor agonist; SGLT2i, sodium-glucose cotransporter 2 inhibitor.

Supplementary Table 8 Prescription of SMBG test strips in every quarter of 2022, by treatment regimen, among patients receiving the same treatment throughout 2022 and with at least one delivery strips in 2022

| **Current regimen in Q4 2022 (cumulative)** | **Patients with at least one delivery of strips in every quarter of 2022** | **If yes, mean number of strips delivered per day** |
| --- | --- | --- |
| Monotherapy (N = 5206) | 560 (10.8%) | 2.17 +/- 1.03 |
| Metformin (N = 3736) | 355 (9.5%) | 2.09 +/- 0.90 |
| Sulphonylurea/glinides (N = 706) | 103 (14.6%) | 2.40 +/- 1.35 |
| SGLT2 (N = 104) | 15 (14.4%) | 2.39 +/- 1.16 |
| GLP-1 RA (N = 270) | 40 (14.8%) | 2.47 +/- 0.98 |
| DPP4i (N = 366) | 43 (11.7%) | 1.97 +/- 0.99 |
| Other monotherapy (N = 24) | 4 (16.7%) | 2.33 +/- 0.91 |
| Dual therapy (N = 5578) | 754 (13.5%) | 2.13 +/- 0.94 |
| Metformin + Sulphonylurea /glinide (N = 1235) | 159 (12.9%) | 2.27 +/- 1.21 |
| Metformin + DPP4i (N = 2058) | 225 (10.9%) | 2.02 +/- 0.73 |
| Metformin + GLP-1 RA (N = 1114) | 187 (16.8%) | 2.19 +/- 0.98 |
| Metformin + SGLT2 (N = 412) | 53 (12.9%) | 2.06 +/- 0.82 |
| Sulphonylurea/glinides + DPP4i (N = 336) | 68 (20.2%) | 2.00 +/- 0.70 |
| Sulphonylurea/glinides + GLP-1 RA (N = 213) | 36 (16.9%) | 2.12 +/- 0.98 |
| Sulphonylurea/glinides + SGLT2 (N = 77) | 10 (13.0%) | 2.52 +/- 0.88 |
| SGLT2 + GLP-1 RA (N = 46) | 7 (15.2%) | 2.07 +/- 0.76 |
| Other dual therapy (N = 87) | 9 (10.3%) | 2.25 +/- 1.00 |
| Triple therapy (N = 3745) | 617 (16.5%) | 2.18 +/- 0.95 |
| Metformin + Sulphonylurea/glinides + DPP4i (N = 1775) | 265 (14.9%) | 2.07 +/- 0.89 |
| Metformin + Sulphonylurea/glinides + GLP-1 RA (N = 1009) | 212 (21.0%) | 2.26 +/- 0.99 |
| Metformin + Sulphonylurea/glinides + SGLT2 (N = 278) | 39 (14.0%) | 2.22 +/- 0.85 |
| Other triple therapy (N = 683) | 101 (14.8%) | 2.25 +/- 1.03 |
| Other multi-therapy (N = 641) | 101 (15.8%) | 2.27 +/- 1.71 |
| Insulin regimens (N = 10395) | 4689 (45.1%) | 2.80 +/- 1.23 |
| Basal Insulin+/- GLM wo pump (N = 6091) | 2873 (47.2%) | 2.51 +/- 1.05 |
| Multi-injection +/- GLM wo pump (N = 3966) | 1712 (43.2%) | 3.26 +/- 1.35 |
| Pump (N = 217) | 32 (14.7%) | 3.53 +/- 1.70 |
| Other insulin therapy (N = 121) | 72 (59.5%) | 2.76 +/- 1.16 |
| Treatment not documented (N = 1905) | 184 (9.7%) | 2.52 +/- 0.99 |
| Sulphonylurea/glinides (N = 9500) | 2500 (26.3%) | 2.47 +/- 1.13 |

Data are for patients who maintained the same overall treatment regimen (i.e., always on monotherapy or always on dual therapy, but potentially with changes within those categories) throughout the year 2022.

GLM, glucose-lowering medication; GLP-1 RA, glucagon-like peptide 1 receptor agonist; DPP4i, dipeptidyl peptidase-4 inhibitor; SGLT2i, sodium-glucose cotransporter 2 inhibitor; wo, without.

Supplementary Table 9 Comparison of annual costs between metropolitan France and overseas departments

| **Cost** | **Metropolitan France (N = 76,213)** | **Overseas departments (N = 3608)** |
| --- | --- | --- |
| **Ambulatory costs** | **4,271 € [4,223–4,319]** | **5,330 € [5,090–5,569]** |
| Medication | 1,524 € | 1,467 € |
| Medical auxiliaries  (nursing care, physiotherapy, etc.) | 750 € | 1,725 € |
| Medical devices | 735 € | 767 € |
| Physician consultations  or home visits | 586 € | 572 € |
| Transportation | 258 € | 394 € |
| Dental care | 182 € | 184 € |
| Laboratory tests | 189 € | 191 € |
| Others | 47 € | 30 € |
| **Hospital costs** | **2,213 € [2,158–2,267]** | **1,880 € [1,552–2,209]** |
| ***Total 2022 Costs*** | **6,484 € [6,401–6,566]** | **7,210 € [6,762–7,658]** |
